# The cost-per-QALY threshold in England: Identifying structural uncertainty in the estimates

**DOI:** 10.3389/frhs.2022.936774

**Published:** 2023-01-19

**Authors:** Bernarda Zamora, Adrian Towse

**Affiliations:** ^1^ Department of Surgery and Cancer, Imperial College, London, United Kingdom; ^2^ Office of Health Economics, London, United Kingdom

**Keywords:** health opportunity costs, threshold, parameter uncertainty, structural uncertainty, NHS England

## Abstract

**Introduction:**

There are increasing numbers of estimates of opportunity cost to inform the setting of thresholds as ceiling cost-per-quality-adjusted life year (QALY) ratios. To understand their ability to inform policy making, we need to understand the degree of uncertainty surrounding these estimates. In particular, do estimates provide sufficient certainty that the current policy “rules” or “benchmarks” need revision? Does the degree of uncertainty around those estimates mean that further evidence generation is required?

**Methods:**

We analyse uncertainty and methods from three papers that focus on the use of data from the NHS in England to estimate opportunity cost. All estimate the impact of expenditure on mortality in cross-sectional regression analyses and then translate the mortality elasticities into cost-per-QALY thresholds using the same assumptions. All three discuss structural uncertainty around the regression analysis, and report parameter uncertainty derived from their estimated standard errors. However, only the initial, seminal, paper explores the structural uncertainty involved in moving from the regression analysis to a threshold. We discuss the elements of structural uncertainty arising from the assumptions that underpin the translation of elasticities to thresholds and seek to quantify the importance of some of the effects.

**Results:**

We find several sets of plausible structural assumptions that would place the threshold estimates from these studies within the current National Institute for Health and Care Excellence (NICE) range of £20,000 to £30,000 per QALY. Heterogeneity, an additional source of uncertainty from variability, is also discussed and reported.

**Discussion:**

Lastly, we discuss how decision uncertainty around the threshold could be reduced, setting out what sort of additional research is required, notably in improving estimates of disease burden and of the impact of health expenditure on quality of life. Given the likely value to policy makers of this research it should be a priority for health system research funding.

## 1. Introduction

This paper considers the uncertainty associated with estimating the opportunity cost of health system resources in the English National Health Service (NHS). Opportunity cost measures what is given up to adopt or continue funding the use of an intervention, as compared to an alternative use of these resources.

Several estimates of this opportunity cost in the English NHS have been produced by a core group of authors who have evolved their approach over the past decade. These results are summarised in Table 1.

**Table 1:** Estimates of NHS England opportunity cost.

Study	Threshold estimate cost-per-QALY	Year(s) it applies to
Claxton et al. (1, 2)	£18,317	2007/8
Claxton et al. (3, 4)	£12,936	2007/8
Lomas et al. (2019) (5)	£5,000–£15,000	2003/4 to 2012/13
Martin et al. (2021) (6)	£5,000–£10,000	2003/4 to 2012/13

QALY, quality-adjusted life year.

These estimates contrast with the current threshold used by National Institute of Health and Care Excellence (NICE) of £20,000–£30,000 per quality-adjusted life year (QALY) as set out in the NICE Methods Guide (7) and in the agreement between the 2018 UK Department of Health and Social Care (DHSC) and the Association of the British Pharmaceutical Industry (ABPI) (8), and with evidence (9) that, although the median threshold used by NICE is within this range, over 40% of submissions to NICE present incremental cost-effectiveness ratios (ICERs) higher than £30,000.

The studies included in Table 1 are critical of NICE’s continued use of its threshold. Our analysis relates to three of them: Claxton et al*.* (4), Lomas et al*.* (5), and Martin et al*.* (6). A related paper (10) cites the 2013 estimate of £12,936 to argue that “The evidence suggests that more harm than good is being done” [by NICE]. Lomas et al*.* (5) state “The evidence from this article suggests that the NHS's marginal productivity is significantly higher (the cost-per-QALY is significantly lower) than that implied by NICE's stated guidance.” Martin et al*.* (6) state that “estimates of marginal productivity in this paper suggest that guidance issued by NICE is likely to do more harm than good, reducing health outcomes overall for the NHS.”

The threshold figures presented in Table 1 are, however, results from an estimation of mean thresholds with several sources of statistical and structural uncertainty. Lowering the threshold without proper consideration of the uncertainty arising from the assumptions used to arrive at these estimates is precisely to risk denying patients access to treatments, using resources for other activities that generate less health gain.

This raises the question as to how certain we can be that the evidence shows that the opportunity cost relevant for NICE decision making is below the £20,000–£30,000 figure. This paper focuses on how structural uncertainty is addressed in the estimation process followed in the studies in Table 1 to get from the regression estimates to a cost-per-QALY threshold. We present alternative estimates derived from different plausible assumptions and methods to illustrate this uncertainty. We also present how heterogeneity of effects different than the mean translate to variability of cost-per-QALY estimates for different clinical areas. Our analysis shows that plausible alternative assumptions indicate that the “central” estimate of the threshold may be within the current NICE range of £20,000–£30,000.

The paper is organised as follows: Section 2 presents types of uncertainty, and the limits to the analysis of structural uncertainty in the three studies. Our results and analyses are presented in Section 3, combining analysis from the studies (4) and (5) with some new plausible estimations. Section 4 deals with heterogeneity, which is different from both parametric and structural uncertainty. Finally, we summarise the implications for threshold estimates, discuss ways in which structural uncertainty could be addressed, and comment on the implications of both structural uncertainty and heterogeneity for research and for policy making related to current estimates of the threshold.

## 2. Handling uncertainty

### 2.1. Types of uncertainty

The Second US Panel on Cost-Effectiveness in Health and Medicine consideration of handling uncertainty in cost-effectiveness analysis (11), reproduced Table 2 in (12) set out below.

**Table 2:** Uncertainty for decision modelling: concepts and terminology.

Preferred term	Concept	Other terms sometimes employed	Analogous concept in regression
Stochastic uncertainty	Random variability in outcomes between identical patients	Variability Monte Carlo error First-order uncertainty	Error term
Parameter uncertainty	The uncertainty in estimation of the parameter of interest	Second-order uncertainty	Standard error of the estimate
Heterogeneity	The variability between patients that can be attributed to characteristics of those patients	Variability Observed or explained heterogeneity	Beta coefficients (or the extent to which the dependent variable varies by patient characteristics)
Structural uncertainty	The assumptions inherent in the decision model	Model uncertainty	The form of the regression model (e.g., linear, log-linear)

Briggs et al. (2012) (12).

We apply this categorisation to the estimates of opportunity cost for NHS expenditure in the three studies (4–6). All three use cross-sectional regression models constructed under the assumption of stochastic uncertainty modelled through an error term which captures unobserved heterogeneity across health units. As a result of the model estimation, parameter uncertainty is reported as the standard deviation (SD) of each coefficient, in particular for the coefficient of interest, which is the elasticity of mortality to health expenditure (termed outcome elasticity). Briggs et al. (12), describe structural uncertainty as including “assumptions inherent to other forms of extrapolation from available evidence, including to other populations and subpopulations and from intermediate outcomes to ultimate measures of health,” and several aspects which include “judgements about the relevance and appropriateness of different sources of evidence.”

All three papers then use the results of these regression models, together with a series of structural assumptions, to estimate a cost-per-QALY threshold.

We briefly set out in the two subsections below firstly, an overview of the model, which uses cross-sectional data, including the reporting of uncertainty; and secondly, an overview of the methods to translate estimations of the relative effects of health expenditure on mortality (outcomes elasticities expressed in percentage points) into an absolute cost-per-QALY threshold (expressed in total incremental health expenditure per QALY), and the reporting of uncertainty around these assumptions.

### 2.2. Uncertainty and the regression model estimation approach

The threshold calculation is built on the use of a cross-sectional data model that uses differences in spending and mortality by 152 geographical units—termed Primary Care Trusts (PCTs) in (4) and local authorities (LAs) in (5) (we use the term PCTs throughout)—subdivided by 23 clinical areas called Programme Budget Categories (PBCs) to arrive at estimates of mortality elasticity, termed “outcome elasticity” for each PBC. Note that, despite the terms “primary” and “local,” these 152 geographical units were, during the period of analysis, responsible for nearly all healthcare expenditure in England, including all hospital care and primary care.

The statistical properties of the model consider health expenditure and mortality to be jointly determined. Estimation of the causal effect of health expenditure on mortality, that is, the estimate of outcome elasticity, has to account for endogeneity bias created by the reverse causation of mortality impacting health expenditure. Methods using instrumental variables (IVs) allow identification of an exogenous source of variation in expenditure. Lomas et al. (5) use IVs from the same set of socioeconomic variables as Claxton et al. (4), using UK census data for 2001 and 2011. The choice of IVs is based on testing statistical properties that show relevance as predictors of health expenditure, and validity as affecting mortality only *via* their effect on health expenditure. These socioeconomic variables are related to needs and deprivation variables considered in the definition of the “funding rule” used to allocate NHS money equitably to different parts of the country (13). Some variables defining the funding rule have also been used to identify the effect of health expenditure under a very different approach in (6), the third of the three papers we are discussing. This builds on (13,14), and its use by (6) implies a more fundamental change in the econometric model: a different health expenditure variable using total NHS spend instead of individual PBC spend, where total NHS spend is the aggregate across all 23 PBCs. The different health expenditure variable creates model uncertainty, a separate source of structural uncertainty from the statistical uncertainty arising from the choice of IVs.

All three papers discuss stochastic and parameter uncertainty around the regression model, as defined in Table 2. It is not in the remit of this paper to discuss uncertainties arising from the regression model any further save to make two points.

#### 2.2.1. Heterogeneity

None of the studies report on the implications of heterogeneity derived from variability in outcomes for identical patients or within each clinical area. This variability is reflected as differences across geographical areas. To account for heterogeneity in mortality, quantile regression (QR) methods have been applied by (15) and also by (16) to examine the impact of expenditure on mortality across the mortality distribution rather than only at the mean.

We consider the implications of both types of heterogeneity in Section 4.

#### 2.2.2. Impact of small sample size on joint estimation

Although the data used in (4, 5) have a panel structure of 152 PCTs followed for 10 time periods, the small sample size of 152 prevented the joint estimation of the model for all PBCs and the model has been estimated separately for each PBC and each year. The separate estimation of the model caused a shortfall in the estimation of the change in health expenditure, which was circumvented with an additional assumption, which is a source of structural uncertainty. In (5), the authors adjust upwards the estimated spend elasticities in the same proportion *k* (not reported in the article). In (4), the adjustment implies *k* *=* *1.38* for 2008–2009. This assumption allocates the shortfall in total spend across all PBCs proportional to their estimated expenditure elasticities. An Office of Health Economics (OHE) report (17) points out the sensitivity displayed by the threshold estimate to plausible alternative assumptions as to the allocation of the missing or underestimated expenditure. Indeed, were the expenditures not to be reallocated, as in the first two Centre for Health Economics Research Paper (CHERP) 81 reports (1, 2) the “central” estimate of the threshold is £18,317, not £12,936, as shown in Table 1.

### 2.3. Uncertainty in moving from regression outputs to estimating a cost-per-QALY threshold

The next challenge, which is the main focus of this paper, is moving from the estimation of outcome elasticities to a cost-per-QALY estimate at health system level, the incremental healthcare cost of producing an incremental QALY. In the context of an overall NHS estimate, it is obtained as the following ratio:

(1) $$\mathrm{Cost}\;\mathrm{per}\;\mathrm{QALY}=\frac{\left(\mathrm{The}\;\mathrm{absolute}\;\mathrm{change}\;\mathrm{in}\;\mathrm{expenditure}\;\;\mathrm{from}\;\;a\;z\%\;\mathrm{NHS}\;\mathrm{budget}\;\;\mathrm{increase}\;\mathrm{summed}\;\;\mathrm{for}\;\mathrm{all}\;\mathrm{PBCs}array\right)}{\left(\mathrm{The}\;\mathrm{absolute}\;\mathrm{change}\;\left(\mathrm{reduction}\right)\;\mathrm{in}\;\mathrm{the}\;\mathrm{QALY}\;\mathrm{burden}\;\mathrm{associated}\;\mathrm{with}\;\mathrm{expenditure}\;\mathrm{change}\;\mathrm{summed}\;\;\mathrm{for}\;\mathrm{all}\;\mathrm{PBCs}\right)}$$

The numerator in Equation 1 can be any assumed absolute change of NHS budget. Lomas et al*.* (5) assumed £10 million, whereas Claxton et al*.* (4) assumed 1% of total NHS budget. The denominator is calculated for each PBC using estimations from the econometric model combined with the structural assumptions, with total QALY change derived by aggregating estimated gains for each PBC.

Estimation of the denominator is based on the econometric model for each PBC, as discussed in Section 2.2, which estimates the effects of spending on mortality in terms of outcome elasticity [percentage change in standardised years of life lost rate (SYLLR) for a 1% change in PBC spend]. The denominator accounts both for spend and outcome elasticities, and the estimation of the “QALY burden” of disease, to arrive at the estimated change in QALYs attributed to any assumed *z*% change in PBC spend. We now analyse the structural assumptions involved in moving from estimating outcome elasticities to a cost-per-QALY threshold for the NHS.

In order to analyse the sources of structural uncertainty in estimating an absolute threshold, we follow (18) in labelling the three major sets of assumptions that underpin this part of the analysis, as

1. Duration: the relationship between expenditure in year *t*, *t* + 1, etc. and health in year *t*, *t* + 1, etc. This is key to translating expenditure into a death averted and additional life years.
2. Surrogacy: converting an estimate of mortality into one of QALYs for each of the 11 PBCs reporting mortality data, in particular estimating the impact of expenditure to improve morbidity rather than mortality. Many assumptions and evidence from different sources are needed to translate mortality reduction into QALY gains.
3. Extrapolation: moving from the 11 out of 23 PBCs reporting mortality data to estimating QALY effects in those without mortality data.

Of the three reviewed studies, only (4) estimates some of the potential effects of structural uncertainty associated with this final, key, stage of the analysis. The choice by (4) of a preferred method to estimate absolute QALYs, namely, the QALY burden method, and presentation of a particular combination of assumptions in scenarios, leads to a “best estimate.” This method and supporting assumptions are then used in both (5) and in (6). Lomas et al*.* (5) refer to the surrogacy and extrapolation assumptions, referencing (4), and quoting Soares et al*.* (19) as evidence of the plausibility of these assumptions. In (5), the only explicit reference to structural uncertainty is in relation to the IV strategy in the regression model. Martin et al. (6). also do not use the term “structural uncertainty.” They discuss methods to calculate the overall QALY disease burden, and refer to Soares et al*.* (18) to support the surrogacy and extrapolation assumptions needed to apply the elasticities to the disease burden.

As a consequence, the reporting of the importance of the structural uncertainty arising from these assumptions in the three papers is limited:

• Claxton et al. (4) report the uncertainty of the estimated threshold as parameter uncertainty, since only parameter uncertainty is used to obtain a probability distribution of the system-wide threshold [Figure 5 page 79 in (4)]. This uncertainty is modelled from the estimated SD of the elasticities of the econometric model for the clinical areas with mortality outcomes. In other words, the reported uncertainty is only based on simulating a normal distribution for the estimated outcome elasticities, where this normal distribution has the estimated means and SDs as parameters. This distribution results in an 89% probability that the threshold is below £20,000 and a 97% chance of it being below £30,000.
• Similarly, both (5) and (6) use Monte Carlo simulations for the normal distribution of each estimated outcome elasticity to obtain a 95% Confidence Interval (CI) for the system-wide threshold. Lomas et al. (5) report 90% CIs of £11,812–£19,861 for 2012/13 (although the range for the full 10-year period is £4,110–£32,881). Martin et al. (6) report 95% CIs of £4,200–£22,300, and compare graphically these CIs with those obtained in (5).

Given that the structural uncertainty in moving to threshold estimates is not parametrised under a probability distribution, an assessment of each element of this structural uncertainly is essential. Without it, we have a non-transparent link between the econometric analysis and the cost-per-QALY threshold results. We now discuss and critically analyse the three key areas of structural uncertainty involved in moving from the regression outputs to an absolute cost-per-QALY threshold.

## 3. Three structural and model assumptions

### 3.1. The duration assumption

The use of a static model and related assumptions about the duration of life expectancy and quality of life of “deaths averted” are key to the model estimates and to the Claxton et al*.* (4) assumptions of structural uncertainty. We analyse the duration assumption, firstly, related to static vs. dynamic models and, second, in relation to the structural assumptions made by (4).

#### 3.1.1. The use of a static model

A contemporaneous relative effect on health gain can be applied either to lifetime health or to health gain measured according to 1 year of disease. In a static econometric model of the type used in the three studies, the duration of effect can only be a contemporaneous effect that measures the relative effect on mortality reduction in 1 year. As Claxton et al*.* (4) state *“Health effects of changes in 1 year of expenditure are restricted to 1 year*.*”* Long-run effects can only be defined in a dynamic model.

Clearly, health expenditure in year *t* impacts health in years *t* *+* *1, t* *+* *2*, etc., as well as in year *t*. But health in year *t* depends not only on health expenditure in year *t*, but on year *t* *−* *1, t* *−* *2*, etc. We can reword the (4) study's assumption as *“All changes in health effects occurring in a particular year are assumed to be the result of changes in expenditure in that same year”.* The static nature of the expenditure and outcome models impose this assumption, however worded. It is an important simplifying assumption, but one which has analytical consequences.

The static econometric model for the mortality outcome is represented as

(2) $$h_{i}=\gamma_{0}+\gamma_{1}n_{i}+\gamma_{2}x_{i}+\varepsilon_{i}$$

where the dependent variable is the observable mortality at a PCT level measured in logarithms as

$$h_{i}=\mathrm{standardised}\;\mathrm{years}\;\mathrm{of}\;\mathrm{life}\;\mathrm{lost}\;\mathrm{rate}\;(3\;\mathrm{year}\;\mathrm{average})$$

The SYLLR represents the potential years of life lost if the population of England and Wales had the same population structure as the European Standard Population. The SYLLR is used to adjust mortality rates so that deaths at younger ages are weighted more heavily than those at older ages. SYLLR data are available for different health locations from ONS mortality statistics. The cause of death by International Classification of Diseases (ICD) is not available for all PBCs. Therefore, the model in Equation 2 has only been estimated for 11 PBCs.

The outcome model parameter of interest is $\gamma_{2}$, the outcome (mortality) elasticity coefficient of PBC expenditure in logarithms $\left(x_{i}\right)$. The effect is controlled for the level of need $(n_{i})\;$ in the PBC clinical area. The outcome model is estimated separately for each year, and for each PBC for which mortality data are available.

An expenditure model is also estimated for each one of the 10 years and for each of the 23 PBCs that serves to allocate total NHS expenditure among PBCs. This expenditure model is represented as

(3) $$x_{i}=\beta_{0}+\beta_{1}n_{i}+\beta_{2}m_{i}+\beta_{3}y_{i}+\varepsilon_{i}$$

where the log of PBC expenditure $x_{i}\;$ is a function of the logarithm of the total PCTi budget $\left(y_{i}\right)$, needs in the PBC $\left(n_{i}\right)$ and in the rest of the PBCs $\left(m_{i}\right)$. The parameter of interest is the expenditure elasticity $\beta_{3}$, i.e., the estimated percentage by which a 1% change in total NHS expenditure changes expenditure in a particular PBC.

##### 3.1.2. Implications of a dynamic model

Had the researchers used a dynamic model, this would have had different implications for estimating the duration of the effects over time. For example, consider a typical dynamic effects distributed-lag model with an outcome equation that allows effects of past expenditure on current mortality, and assumes that the effect decreases at a rate of $0<\lambda <1_{.}$

(4) $$h_{i,t}=\gamma_{0}+\gamma_{1}n_{i,t}+\gamma_{2}x_{i,t}+\gamma_{2}\lambda x_{i,t-1}+\cdots +\gamma_{2}\lambda^{k}x_{i,t-k}+\varepsilon_{i,t}$$

Note that the first-order autoregressive lag model can be written in an infinite-distributed-lag form, with k=∞, in the model shown above (Equation 4). This model accounts for the inertia of past mortality and is expressed as

(5) $$h_{i,t}=\delta_{0}+\lambda h_{i,t-1}+\gamma_{1}n_{i,t}+\gamma_{2}x_{i,t}+\varepsilon_{i,t}$$

In a dynamic model such as this one there is a contemporaneous or short-run effect measured by the elasticity $\gamma_{2}$. This effect captures the impact of current changes in health expenditure on mortality, assuming that past health expenditure is unchanged.

(6) $$\gamma_{2}=\frac{\Delta h_{i,t}}{\Delta x_{i,t}}$$

The dynamic effects distributed-lag model (Equation 4) corresponds to the lag weights of the (possibly) infinite moving-average representation and requires that the relationship between mortality and health expenditure be stationary which implies that coefficient $\left(\gamma_{2}\lambda^{k}\right)$ measures either the effect of current expenditure $\left(x_{i,t}\right)$ on future mortality $\left(h_{i,t+k}\right)$ or the effect of past expenditure $(x_{i,t-k})\;$ on current mortality $\left(h_{i,t}\right)$. For each lag *k*, these effects capture the dynamic marginal effects of temporary changes of health expenditure on mortality with different delays/lags, always assuming a temporary change of health expenditure to a given level in previous periods (20).

The long-run effect that accounts for the inertia could be captured in the finite distributed-lag model which approaches the long-run effect estimated in a dynamic model with lagged mortality as explanatory variable as the model:

(7) $$\overset{k}{\underset{\;j=0}{\sum }}\frac{\Delta h_{i,t}}{\Delta x_{i,t-j}}=\overset{k}{\underset{\;j=0}{\sum }}\gamma_{2}\lambda^{j}=\gamma_{2}(1+\lambda +\lambda^{2}+..+\lambda^{k})\approx \frac{\gamma_{2}}{1-\lambda }$$

The long-run effect captures the effect of a permanent increase of health expenditure in the current year, and it assumes that this increase is kept in all future periods. This is also called the long-run cumulative effect.

Lomas et al. (5) and Martin et al. (6) reference Soares et al. papers (18, 19) reporting on an elicitation study as providing support for the structural assumptions necessary to move from the regression elasticities to a cost-per-QALY threshold to be described as “conservative.” In seeking to parameterise this structural uncertainty, (18) elicits the beliefs of experts about the magnitude of effects in the second, third, and fourth years after the change in expenditure. That is, according to the dynamic effects distributed-lag model (Equation 4), they aim at measuring the effect:

(8) $$\overset{3}{\underset{\;j=0}{\sum }}\frac{\Delta h_{i,t}}{\Delta x_{i,t-j}}=\gamma_{2}(1+\lambda +\lambda^{2}+\lambda^{3})$$

The experts were asked in (18) to express an opinion on the rate λ, the proportion of the effect on successive years from a change in expenditure in the first year. This proportion is, in principle, estimatable and can vary across years. In this case the stationarity assumption of the distributed-lag model, with decreasing effects, may not hold. More advanced econometric models could account for the non-stationarity of the relationship. In a dynamic model, the contemporaneous effect is smaller in absolute value than the medium-term or long-run effect $\left(|\gamma_{2}|<\;|\gamma_{2}(1+\lambda +\lambda^{2}+\lambda^{3})|\right)$. However, this inequality holds if $\gamma_{2}$ is estimated from a dynamic model, not necessarily when comparing a static model with a dynamic one. Therefore, the elicitation question posed by Soares at al. (18) is not a reasonable interpretation of (4–6), since the estimation of the outcome elasticities from static and dynamic models cannot be compared. Thus, the approach of (18) implies that it is reasonable to add on the health effects for future years from today's expenditure, but not to deduct the health effects occurring today that arise from previous years’ expenditure. The paper concludes that “mortality effects are expected also to occur in subsequent years. This suggests that the original work underestimated the QALY impacts of changes in expenditure”. The paper is, however, using an asymmetrical approach which will overestimate the health gains obtained from current year expenditure.

This brings us back to the question of the duration of effect that we are interested in, and whether this is modelled as naïve (ignoring the future) or rational (accounting for today's decisions into the future). The duration of the mortality effect should be defined according to the period of interest for the definition of the opportunity cost of health expenditure, which in the case of all three papers (4–6) is the single fiscal year. This definition matches the definition of immediate/contemporaneous effect defined above. Note that different concepts of short-run and long-run elasticities define different opportunity cost ratios, and different concepts of cost-per-QALY thresholds. In the usual definition, the elasticity that matters is the annual short-run elasticity that can be estimated in either a static or dynamic model.

#### 3.1.3. The Claxton et al. scenarios of structural uncertainty in duration

Because of the use of a static model, Claxton et al*.* (4) need to explore different assumptions about the duration of life years gained (LYG) per “death averted.” It is also necessary to make assumptions about the quality of life in which additional life years are lived. Sources of structural uncertainty are reported in Claxton et al*.* (4), Table 30 in Chapter 5 and Table 179 in Appendix 3. In Chapter 2 (p. 10), the authors state that their estimates are driven by the views taken on two key assumptions on which either an “optimistic” or “conservative” view can be taken:

1. Whether “the health effects of changes in 1 year of expenditure are restricted to 1 year”? Claxton et al. (4) note “this is implicit in the estimates of outcome elasticities estimated in the econometric analysis … [but] is likely to underestimate effects on mortality as expenditure that reduces mortality risk for an individual in 1 year may well also reduce their risk over subsequent years.” The alternative estimate “is based on assuming that health effects are not restricted to 1 year but apply to the remaining disease duration for the population at risk during the expenditure year”;
2. What the mortality risk is of “any death averted by expenditure in 1 year”? The authors’ “optimistic assumption” is that “the years of life gained (LYG) associated with each death averted … will return the individual to the mortality risk of the general population, taking account of their age and gender”. This means that the LYGs per death averted are estimated at 4.5 years.^1^ The “conservative” assumption is “any death averted is only averted for the minimum duration consistent with the mortality data used to estimate the outcome”, i.e., LYGs are restricted to 2.

In combining different estimates of these assumptions the authors find:

• The “best” estimate of the cost-per-QALY threshold of £12,936 comes from taking the “conservative” assumption of restricting health effects to one year, and the “optimistic” assumption of 4.5 years LYG per death averted;
• The “lower bound” of £2,018, comes from the “optimistic” assumption that “health effects are not restricted to 1 year but apply to the remaining disease duration for the population at risk” and the “optimistic” assumption of 4.5 years LYG per death averted;
• The “upper bound” of £29,314 is based on the combination of the “conservative” assumption that health effects are restricted to 1 year, and the “conservative” assumption that “any death averted is only averted for the minimum duration consistent with the mortality data used to estimate the outcome”, i.e., LYGs are restricted to 2.

In relation to the first assumption, as we have noted, restricting health effects to one year is not “conservative” but inherent in the use of a “static” model. The second assumption is that “any death averted by expenditure in 1 year will return the individual to the mortality risk of the general population”. This, as the authors state, is optimistic. In the absence of disease-specific data, there is a clear case for using the assumption that any death averted is only averted for the 2 years duration consistent with the mortality data used to estimate the outcome elasticities and with the implicit assumption of the static model (the future either does not exist or it is ignored). In this case, the threshold would increase, to the Claxton et al*.* (4) “upper bound,” estimated at £29,314.

However, there is a third important assumption which is not addressed in the sensitivity analysis in Table 30 or in the paragraphs quoted above. This is the assumption as to the *quality of life* in which additional years of life are lived by those whose deaths are averted. This is discussed on page 59 using data from 2007. Using “the QoL of the general population is likely to underestimate a cost-per-QALY threshold.” This is contrasted with using the QoL of the original disease state which “is likely to overestimate a cost-per-QALY threshold.” The differences are material. Claxton et al*.* (4) report these differences in their Table 21 for QALYs gained from reduced mortality. The effect of using the QoL relevant to the disease, rather than the general population norm for these LYGs is to increase the threshold by 25.6% in the best estimate, 25.0% in the lower bound estimate, and 24.6% in the upper bound estimate. Why this sensitivity is not shown in the main results reported in Table 30 is not apparent.

In effect, we have an assumption about 1 year health effects, which is inherent in the model and is neither “conservative” or “optimistic”, an assumption of QoL effects during additional LYGs that the authors acknowledge as leading to an underestimate of the threshold (which we can style therefore as optimistic), and then an assumption about additional LYG which is described as “optimistic” as deaths averted return to the mortality risk of the general population. If we were to choose a number in Table 30, following the authors’ logic of combining optimistic and conservative assumptions in the best estimate, it would be logical to combine an optimistic assumption about QoL during LYG (the QoL of the general population, baked into all three of the estimates in Table 30) with the conservative assumption about the number of (2) LYGs. This gives us a best estimate of a cost-per-QALY threshold of £29,314.

We are not suggesting £29,314 is the answer. However, LYGs will be disease-specific rather than at the mortality risk of the general population (i.e., lower than 4.5 years), and QoL during these years will be lower than that of the general population. Thus, the threshold will be above £12,936. We therefore consider that, of the three numbers offered by Claxton et al*.* (4), the one most consistent with the authors’ preference for combining optimistic and conservative assumptions is not £12,936 but £29,314.

##### 3.1.4. Discounting

The static nature of the model, leading to short duration of mortality effects, and an assumption that all morbidity effects not linked to mortality (the structural assumptions behind which we explore below) means that the effect of discounting is relatively small. Claxton et al. (4) report that “although this estimate of £12,936 reflects changes in undiscounted QALYs associated with changes in expenditure, discounting the QALY effects only increases the cost-per-QALY threshold to £13,141.” This is an increase of 1%. If, as we discuss above, the assumption of only 2 additional LYGs rather than 4.5 was used, the effect would be even smaller. Thus, the static nature of the model allows us to ignore discounting.

### 3.2. The surrogacy assumption

As we have noted, a surrogacy assumption is needed to perform the translation from SYLLR mortality effects in those PBCs reporting mortality to a change in QALYs that takes in to account QALYs generated from QoL improvements that do not arise from averted deaths. Unfortunately, EQ-5D or some other generic measure of health-related quality of life is not routinely collected by the NHS, thus it is not possible to independently estimate the QoL benefits of health expenditure. We divide our discussion of the surrogacy assumption into two parts. In the next subsection, we explore the choice of “QALY burden” rather “QALY ratio” as the basis for estimating disease burden, which has the effect of reducing the threshold estimate, and in the later subsection we discuss the assumption of perfect surrogacy of quality-of-life effects, i.e., that the NHS is as good at reducing the burden of morbidity as it is at reducing mortality.

#### 3.2.1. QALY ratio vs. QALY burden approaches

The surrogacy assumption used in all three studies assumes mortality effects $\gamma_{2}\;$ are estimated for each clinical area using a *QALY burden method*. This assumption implies that the outcome elasticity obtained in the mortality outcome model can be used as a surrogate effect to calculate the more complete measure of health effect of absolute QALY gains for each PBC. This measure of QALY burden is affected by assumptions and data availability, which we discuss below.

• The change in QALYs arising from reduced mortality is termed, by Claxton et al. (4), change in QALYdeath;
• The change in QALYs from reduced morbidity not related to any change in mortality but more generally to the impact of expenditure in a disease area on the QALY burden during disease is termed change in QALYalive.

In the QALY ratio method, the relevant effect is the reduction in years of life lost (YLL) according to the mortality effect. To obtain the QALY gain, the reduction in YLL is multiplied by the ratio of the total QALY burden in a given PBC over the mortality burden measured in YLL.

The surrogacy assumption consists of moving from the effect of health expenditure on mortality, $\gamma_{2}$, (leading to change in QALYdeath) to the effect on QALY gains from tackling morbidity (change in QALYalive). We discuss below how the surrogacy assumption impacts the estimation of the cost-per-QALY threshold in (5) using the example of the estimation of the cost-per-QALY for PBC2, cancer.

**Figure 1:**
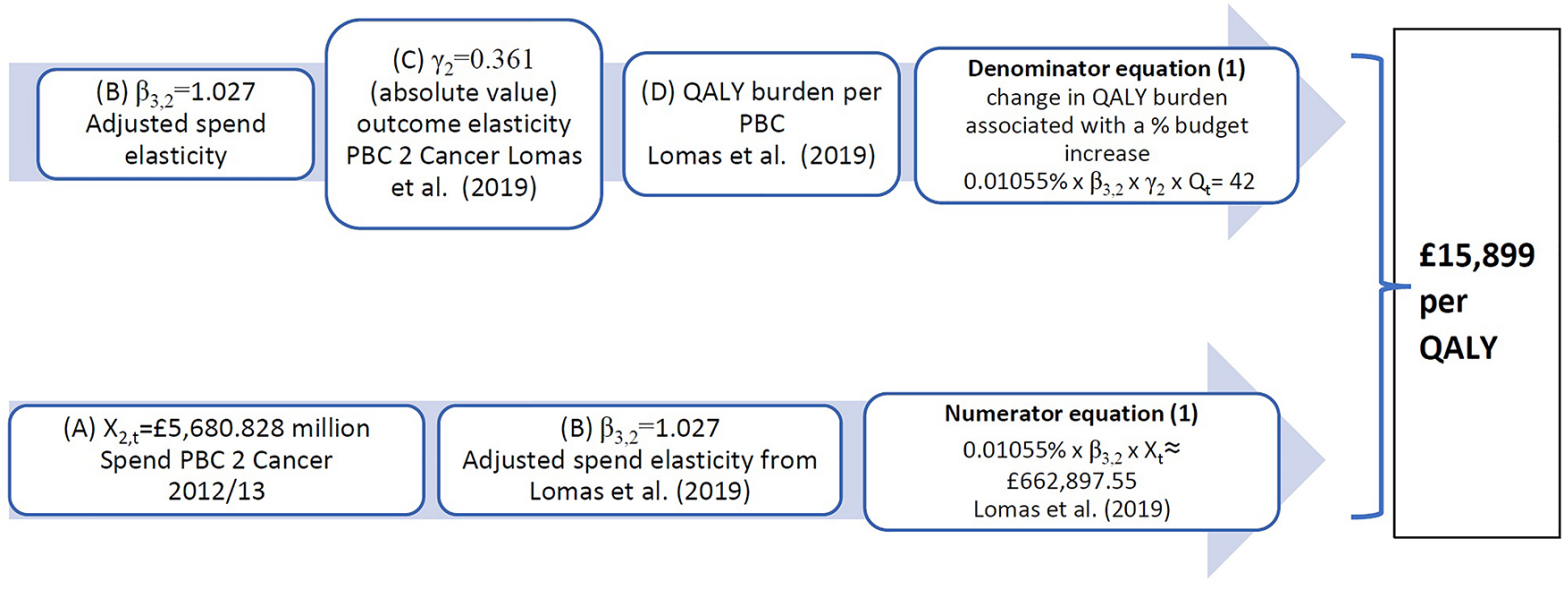
Calculation of implied PBC cost-per-QALY for cancer. QALY, quality-adjusted life year; PBC, Programme Budget Category.

Given that mortality is only observed for 10 PBCs in the three studies, the outcome model (Equation 2) is only estimated for each of these 10 PBCs [there are 11 but maternity and neonates are aggregated into one in (5)], and outcome elasticities $\hat{\gamma_{2,k}}$ for the last period are estimated from expenditure of 2012/13 and the mortality rate (SYLLR) for the period 2012–14. Taking the example of cancer, we have an expenditure elasticity $\;{\hat{\beta }}_{3,2}=1.027;$ and an outcome elasticity $\hat{\gamma_{2,2}}=0.361.\;$ Lomas et al. (5) present overall estimates for the NHS of the cost-per-QALY from an assumed change of £10 million in the NHS budget. For 2012/13 this represents a 0.01055% increase in the NHS budget. Figure 1 illustrates how these estimates could have been obtained from the cancer PBC, using additional data obtained from the York Team (21) research project supporting the study (5) (summarised in Supplementary Appendix S1, Table A3) and from (15). In particular, we highlight the sensitivity to two types of assumptions:

• the assumed change in PBC expenditure and
• the calculated or implied total QALY burden.

Taking estimated spend, outcome elasticities, and change in QALY burden from (5), the implied total QALY burden for cancer $Q_{2,t}$ could be estimated and gives $\hat{Q_{2,t}}=1,073,707.4$ QALYs.

In (4), the cancer PBC has implied cost-per-QALY for 2008 of £16,997, which results from a spend increase of £35 million and a QALY gain of 2,064 QALYs. This implies a QALY burden for cancer of about 928,609 QALYs in total for all cancer patients in England, made up of QALYs that can be gained from reducing mortality weighted by their quality of life (QALYdeath) (93% of QALYs) and pure QALY gains that can be obtained by improving quality of life for those who do not die (QALYalive) (7% of QALYs). These percentages are similar in (5), where the 42 QALYs gained from £663,000 PBC spend is distributed as 93% of QALYs (39 QALYs) from avoiding premature deaths and 7% QALYs (3 QALYs) as QALYalive, gained from reducing morbidity during the disease.

Supplementary Appendix S1 summarises the QALY ratio and QALY burden methods highlighting the important differential effect of these approaches for the QALY distribution across clinical areas which impacts the overall threshold estimate. The QALY burden method better accounts for QoL during disease for PBCs with mortality (e.g., respiratory) but worse account of QoL for PBCs without mortality (e.g., mental health). This has implications for the reporting in Claxton et al.’s (4) Table 30 of lower thresholds for the “big four” programmes and for the “11 PBCs with mortality.” These thresholds would be quite different and higher if the QALY ratio method had been used. In summary, the impact on the threshold reported in (4) is that the cost-per-QALY in PBCs for 2006/7 is 14% higher at £11,638 using the QALY ratio, than at £10,187 using the QALY burden. The results are not reported for 2007/8.

The main argument for using the QALY burden approach is that, with the ratio approach “much of the information that is available about the other 13 PBCs [without mortality data] cannot be used to inform the estimates of the cost-per-QALY threshold” [(4), p. 65]. We do not agree. We cannot escape from the absence of mortality data for these 13 PBCs, when mortality elasticities from the regression model are the drivers of the overall threshold estimates. The other fundamental difference in the calculation of QALY change for PBCs without mortality as between the two approaches is the extrapolation method which we discuss below. In the QALY burden method, a weighted average of the mortality elasticities is applied directly to the QALY burden for the PBCs without mortality data. This gives the appearance of simplicity to what is, in reality, a series of major assumptions, all of which give rise to structural uncertainty.

#### 3.2.2. Assumption that the impact of expenditure on quality of life is proportionate

To illustrate the effect of the surrogacy assumption, assume that all of the effect of health expenditure is on reducing mortality and so to the LYGs and to the QoL obtained during these extra LYs. We again follow Claxton et al*.* (4) in terming these QALYdead. That is, there is no effect from health expenditure on morbidity reduction from those whose deaths are not averted in the time period. In this case, the cost-per-QALY threshold would increase by 7.5% for cancer, i.e., the PBC cost-per-QALY for cancer would increase by a ratio of 1/0.93, from £15,898 to £17,095, given that the part of QALY burden gained from reducing premature deaths represents 93% of the total change in the QALY burden. Cancer has far and away the highest mortality share of QALY burden, so for all other disease areas the impact would be larger. Of course, it is not at all appropriate to suggest there are no “pure” QoL QALY gains (QALYalive) in any disease area. The point is to illustrate the sensitivity of the QALY burden estimate and the surrogacy assumption as we move from a cost-per-QALYdead to a cost-per-QALY gain, where the QALYs gained are (QALYdead + QALYalive). As shown in Supplementary Appendix S1 Table A3, taken from the analysis underpinning Lomas et al*.* (5), of the total of 694 QALYs estimated to be gained from an increase in overall NHS expenditure of £10 m, 500 (72%) are “pure” QoL (QALYalive), i.e., they depend on the surrogacy assumption.

All the threshold estimates “assumed perfect surrogacy” (18). As the OHE report (17) puts it, this assumes that “PCTs are as good at improving quality of life, which we cannot observe, as they are at reducing mortality, which we can.” Yet, as (17) set out there are good reasons for thinking this is not the case. Firstly, PCTs contract for services that achieve things other than QALY maximisation. They are required to target reductions in waiting times and non-QALY-related activities that are important to decision-makers and subject to targets. For a discussion of factors other than QALY gains that the NHS sees as important see (22). Most will have some health impact—certainly in the case of waiting list reduction—but this is not the main reason for them. These take resources away from the pursuit of QALY maximisation from a fixed budget. Secondly, given the priority given to reducing mortality in the NHS, it is likely that a lower priority is given to addressing disease that primarily impacts quality of life, particularly as it is not being routinely measured. QoL improvement targets feature in the second of five domains of the NHS Outcomes Framework (23) “Enhancing quality of life for people with long-term conditions.” However, QoL is only measured with EQ-5D collected in primary care. Domain 1 is Preventing people from dying prematurely. It is a hierarchy with rehabilitation, a positive experience of care, and protecting patients from avoidable harm making up the remaining domains. The effect of assuming less than “perfect surrogacy” is that increases in QALYdeath do not lead to proportionate increases in QALYalive.

A second important aspect is whether all of the morbidity burden can be reduced by health interventions? We can look at the US study (24), which estimated “QALYs lost due to death” using similar processes to those used in these three papers. However, they go on to say that “we estimated QALYs lost due to morbidity assum [ing] that 10% of morbidity is amenable to health care. We further assumed … the same proportional effect on amenable morbidity as …. on mortality.” Proportionality was applied to only 10% of the morbidity burden. The basis for the 10% estimate is a paper by Kaplan and Milstein (25). We are not aware of a similar paper looking at the UK population.

Table 3 presents different assumptions on the proportionality of QoL gains to mortality effects. These differences may arise from less focus on improving quality of life or that less of the burden of morbidity is amenable to being impacted by healthcare interventions. The proportionality assumption is represented by column 1 from (5). Columns 2 and 3 decrease the proportionate impact on QoL. Column 4 is the upper limit assuming no QALYalive gains from reducing morbidity.

**Table 3:** Summary of overall cost-per-QALY with different surrogacy assumptions.

	Perfect surrogacy all PBCs (except 22 and 23) QALY change = QALY death + QALY alive	PBCs with mortality outcomes: QALY change = QALY death + QALY alive PBCs without mortality outcomes No “pure” QoL effects QALY change = QALY death	PBCs with mortality outcomes: QALY change = QALY death +50% QALY alive PBCs without mortality outcomes 50% QoL effects	All PBCs (except 22 and 23) No “pure” QoL effects QALY change = QALY death
Extrapolation PBCs without observable mortality $\bar{\gamma_{2}}=1.15$ Lomas et al. (5)	£14,410	£19,231	£22,497	£51,546

QALY, quality-adjusted life year; PBCs, Programme Budget Categories.

Table 3 also presents a summary of the effects of the surrogacy assumption.

In Supplementary Appendix S2 Figure A1, we show the threshold points corresponding to different surrogacy assumptions as presented in Table 3. These points illustrate that the percentage increase in the threshold is far larger than the percentage decrease in QALYs. This figure is not dissimilar to that included in Claxton and Sculpher (26), with the important exception that, if we want to fit an “elasticity of the threshold” curve, it needs to go through the outcomes of the relevant scenarios from the analysis of the structural uncertainties.

Soares et al*.* (18) argue that their expert elicitation exercise shows that “surrogacy is expected to be greater than 1 (this holds across disease areas for the first, second, and third years), indicating that the effects of changes in expenditure on total QALY burden are, in proportionate terms, expected to be higher than (rather than equal to) those on mortality burden. Again, this suggests that the original work underestimated the QALY impacts of changes in expenditure.” Whilst it is quite plausible that spending to reduce morbidity in year t has more effect in years *t* + 1, *t* + 2, and *t* + 3 than spending to reduce mortality, this is irrelevant in the static model that the authors use to estimate the impact in year *t*. Their elicitation exercise shows that the impact on quality of life in year *t* (the focus of the analysis) depends on spending in earlier years and is not solely attributable to spending in year *t*.

We do not have the data to make a plausible estimate of the surrogacy effects. However, the relative importance of mortality reduction in NHS priorities, as compared to QoL improvement, and the evidence from the US that as little as 10% of morbidity may be potentially ameliorated by healthcare intervention, suggests that, of the numbers set out in Table 3, a threshold of £22,497, in which the effectiveness of marginal non-mortality reduction expenditure is assumed to result in an increase in QALYs from QoL improvements that is, at the margin, half that of those achieved by mortality reduction, may well be closer to reality than a threshold that assumes perfect surrogacy. This is an area where further work is required.

### 3.3. The extrapolation assumption

An extrapolation assumption is needed because Equation 2 can be only estimated for 10 of the 23 PBCs, those with observable mortality rates data at health location level. The extrapolation assumption is used to impute the health effect estimated for these 10 PBCs to the rest of the PBCs.

As we set out below, different assumptions about extrapolation impact the threshold. Our analysis shows that an alternative plausible extrapolation method increases the threshold for non-observed mortality PBCs from £27,089 to £43,079, an increase of nearly 60%. The impact on the overall threshold is, of course, much lower, increasing it by 10%, because of the larger weight of the observed mortality PBCs within the change in QALY burden surrogacy measure.

As noted, Claxton et al*.* (4) use two different extrapolation methods for different measures of QALYs:

(1) if the QALY ratio approach is used, then extrapolation projects the ***absolute*** average cost-per-QALY obtained for the 10 PBCs with observable mortality to the rest of PBCs;
(2) if the QALY burden method is used, extrapolation considers the effect of total expenditure on the ***relative*** health gain (outcome elasticity) obtained for the 10 PBCs with observable mortality to the rest of PBCs. The three papers, (4–6), all focus on this second extrapolation method. Lomas et al. (5), for example, estimate a weighted average as elasticity of extrapolation $\left(\bar{\gamma_{2}}\right)$ using outcome elasticities and spend data from the 10 PBCs with observable mortality. This constant elasticity of extrapolation is then applied to all the PBCs without mortality data for which it is assumed that health expenditure has an effect on health gain (i.e., 10 out of the 12 PBCs without mortality data).

The method used in (5) to obtain a constant elasticity of extrapolation which imputes the outcome elasticity for PBCs without observable mortality, estimates an elasticity for extrapolation $\bar{\gamma_{2}}=1.15$, as reported in (21). The York Team also look at a lower elasticity of extrapolation at $\bar{\gamma_{2}}=0.79$. Both elasticities of extrapolation are calculated as different weighted averages of the estimated outcome elasticities. Lomas et al*.* (5) adds an adjustment according to the mortality level of the PBC, while (4) only accounts for the level of PBC spend.

We analyse how different elasticities of extrapolation can be estimated for each PBC without mortality outcomes, instead of a single elasticity of extrapolation as used by both (4) and (5). The definition starting point is the same. The total change in NHS expenditure used for (5) calculations is £10 million. Of this, £4.934 million (just under half) is allocated by the expenditure elasticities to be spent on the 11 PBCs with mortality data. The total change in QALY burden for these 11 PBCs is 507 QALYs, which corresponds to an implied total QALY burden for these 11 PBCs of 6,695,925 QALYs. If we consider the definition of proportionate effect as explained in Figure 1, this results in an average relative QALY gain of $(\bar{\beta_{3}\times \gamma_{2}})=0.719.$ However, although the spend elasticity $\beta_{3}$ has been estimated for all PBCs, this average relative QALY gain of 0.719 is not used by (5) to obtain an elasticity of extrapolation for the PBCs where mortality is not observed. Using Lomas et al*.*’s (5) spend elasticities $\beta_{k,3}\;$ presented in Supplementary Appendix S1 Table A3, we obtain elasticities of extrapolation for each PBC *k* without mortality data as

$$\gamma_{k,2}=\frac{\left(\bar{\beta_{3}\times \gamma_{2}}\right)}{\beta_{k,3}}=\frac{0.719}{\beta_{k,3}}$$

We illustrate the effect of different elasticities of extrapolation for calculation of the implied PBC cost-per-QALY for mental health. Considering the elasticity for extrapolation from Lomas et al*.* (5), $\bar{\gamma_{2}}=1.15$, and the spend elasticity estimated for PBC 5 Mental health $\beta_{5,3}=1.023$ implies that the QALY gain in mental health estimated at 92 QALYs results in £14,289 per QALY [York Team (21)] as reported in Supplementary Appendix S1 Table A3. However, this represents a much larger relative QALY gain for a percentage increase in PBC expenditure than averaging across the PBCs with mortality outcomes, since $\beta_{5,3}\times \bar{\gamma_{2}}=1.023\times 1.15=1.17>(\bar{\beta_{3}\times \gamma_{2}})=0.719.\;$ Similarly, if the elasticity of extrapolation is calculated following Claxton et al*.*’s (4) method with $\bar{\gamma_{2}}=0.79$, the corresponding QALY change in mental health results in 63.2 QALY which implies a PBC cost-per-QALY of £20,748. Both elasticities of extrapolation result in a lower PBC cost-per-QALY as compared to that obtained by using our extrapolation method which calculates $\bar{\gamma_{2}}=(0.719/1.023)=0.70$. Using this elasticity of extrapolation increases the implied PBC cost-per-QALY for mental health from £14,289 to £23,319.

The estimated QALY change for the 12 PBCs without observable mortality ranges from 118 QALYs (using our method for the elasticity of extrapolation) to 187 QALYs [using elasticity of extrapolation from (5)]. As we illustrate in Supplementary Appendix S1 Table A3, QALYs from avoiding premature deaths are 12 for a total spend of £5,065,709 (8 using our method). Thus 93% of the QALY change (175/187) is due to “pure” QoL effects. These figures show the importance of the extrapolation and surrogacy assumptions for these PBCs, which account for 26.9% of the QALY change and just over half of the assumed change of £10 million in NHS budget.

Table 4 summarises the effect of the different assumptions used to calculate the elasticity of extrapolation.

**Table 4:** Implied PBC cost-per-QALY for different elasticity of extrapolation methods.

	Spend elasticity $\beta_{k,3}$	Lomas et al. (5) $\bar{\gamma_{2}}=1.15$	York Team (21) $\bar{\gamma_{2}}=0.79$	Elasticity of extrapolation$\;\gamma_{k,2}:$ $(\beta_{k,3}\times \gamma_{k,2})=0.719$
Disorders of blood	1.119	£7,189	£10,651	£13,094
Mental health	1.023	£14,289	£20,748	£23,319
Learning disability	0	N/A	N/A	N/A
Problems of vision	1.411	£41,341	£59,712	£92,565
Problems of hearing	1.523	£4,510	£6,749	£11,292
Dental problems	0.855	£31,506	£46,080	£43,285
Skin	1.158	£84,740	£134,048	£170,541
Musculoskeletal	0.725	£13,546	£19,420	£15,469
Poisoning and adverse events	1.124	£78,625	£182,443	£225,295
Healthy individuals	1.172	£346,537	£352,784	£454,252
Social care needs	1.613	N/A	N/A	N/A
Other	0.585	N/A	N/A	N/A
12 PBCs without observed mortality	** **	£27,089	£39,434	£43,079
All 23 PBCs	** **	£14,410	£15,714	£15,985

QALY, quality-adjusted life year; PBCs, Programme Budget Categories.

Our assumption for calculating the elasticity of extrapolation for each PBC according to an average relative QALY gain of $(\beta_{k,3}\times \gamma_{k,2})=0.719$ is the most plausible as it aligns with the definition of the extrapolation assumption. This method results in lower elasticities of extrapolation for all PBCs than using the average of $\bar{\gamma_{2}}=1.15$ from (5). Consequently, the resulting PBC costs per QALY are larger for the PBCs affected by the extrapolation assumption, i.e., those without mortality outcomes. As compared to the calculation in (5), our elasticity of extrapolation increases the average cost-per-QALY from £27,089 to £43,079 for the 12 PBCs without observable mortality. However, when considering all 23 PBCs, these differences arising from the extrapolation elasticity have a modest effect of 10% increase in the threshold due to the smaller weight in overall QALY change for the PBCs without observable mortality.

## 4. Heterogeneity

Heterogeneity is another important source of uncertainty identified by Sculpher et al. (11), which is only partially explored by (4), and not at all by (5) or (6). Although heterogeneity is not in itself a structural uncertainty, understanding its importance gives rise to a better understanding of sources of structural uncertainty**.** We consider in the subsections below: firstly, threshold heterogeneity that seems to be driven by differences in mortality rates across geographical locations; and secondly, threshold heterogeneity that depends on the disease being treated.

### 4.1. Heterogeneity across health locations

Within a given clinical area, a quantile regression approach can deal with structural uncertainty from outcomes heterogeneity, with the variation in outcome elasticities analysed and estimated according to the mortality rate of the health location. Research by Hernandez-Villafuerte et al. (15), which includes both authors of this paper, shows that the health effect of health expenditure is determined by the initial level of health. The relationship between the outcome elasticities and the mortality rate of health locations (PCTs) was estimated using quantile regression methods for six PBCs. For five of the six PBCs, the relative effect of health spending on mortality reduction, as measured by outcome elasticities, increases with the mortality rate of the PCT. Consequently, this produces larger QALY changes for PCTs with large mortality rates in these clinical areas and a larger marginal productivity or lower cost-per-QALY for these health locations. The exception was the PBC for infectious diseases, which is related to the epidemiology of the disease.

**Figure 2:**
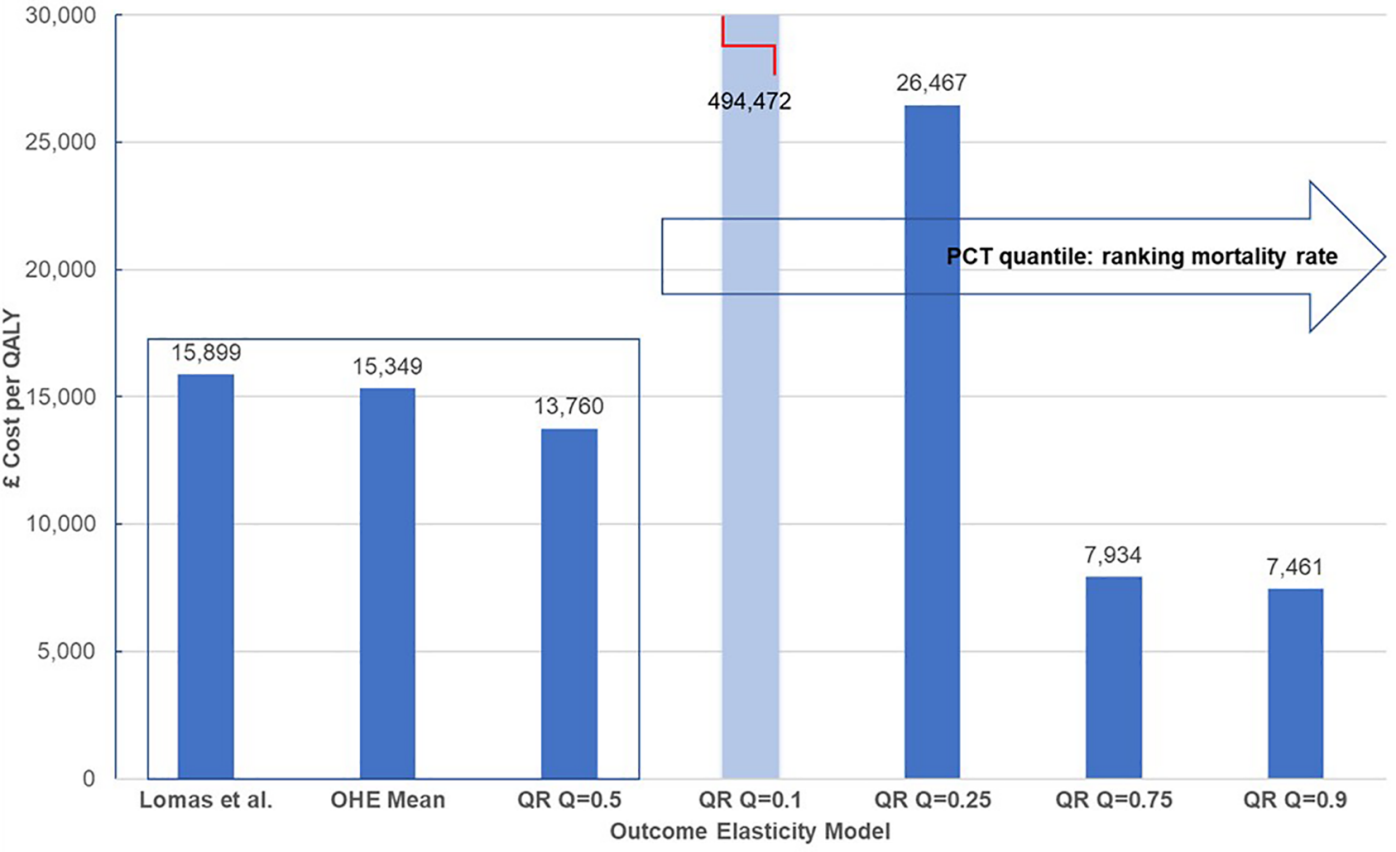
Heterogeneity of cost-per-QALY for cancer. QALY, quality-adjusted life year. Central estimates compare mean estimates from Lomas et al. (5) and the Hernandez-Villafuerte et al. (15) estimation using similar methods, and quantile regression at the median PCT ranked at the mid of mortality rate in England (QR Q = 0.5). Light blue bars indicate that cost-per-QALY is based on a non-significant estimated outcome elasticity.

Table 5 presents outcome elasticities for these six PBCs: infectious diseases, cancer, circulatory, respiratory, endocrine, and gastrointestinal problems for PCTs representing five quantiles in the ranking of mortality rate for each clinical area. The PBC cost-per-QALY is presented for the corresponding outcomes elasticities and the comparison is illustrated graphically for cancer in Figure 2. We show the estimate in (5), and then the estimates in (15) for each quantile, using the same approach as (5), i.e., the paper only adjusts for quantile variation. The rest of the parameters (spend elasticities and implicit QALY burden) are taken from (5). We can see very clearly that outcome elasticities vary according to the quantile of the mortality distribution per PBC. This is different to a simulation from a symmetric distribution such as the normal distribution used in (5). Moreover, the variation in the outcome elasticity is structural and indicates heterogeneity across health locations.

**Table 5:** Heterogeneity of outcome elasticities and cost-per-QALY for six PBCs.

	PBC 1 Infectious	PBC 2 Cancer	PBC 4 Endocrine	PBC 10 Circulatory	PBC 11 Respiratory	PBC 13 Gastrointestinal
Spend (£M) 2012/13 (PBC data)	£1,545.40	£5,680.83	£3,060.55	£6,897.22	£4,693.74	£4,764.42
QALY burden (Lomas et al. (5)]	104,869	1,073,707	639,121	700,301	1,228,713	419,403
Spend. elasticity [Lomas et al. (5)]	0.749***	1.027*	0.951***	1.285***	0.928***	0.997***
Outcome elasticity [Lomas et al. (5)]	0.362***	0.361**	0.499	1.464***	1.704*	1.904*
Outcome elasticity [Hernandez-Villafuerte et al. (15)]	0.3379***	0.3447**	0.2284	1.4678***	1.6957*	1.6963**
QR outcome elasticity Q = 0.5	0.4083**	0.3845*	0.4328*	1.4475***	1.5856*	1.3696*
QR outcome elasticity Q = 0.1	0.7593**	0.0107	0.3593	0.9682**	0.9237	1.2347
QR outcome elasticity Q = 0.25	0.4844**	0.1999*	0.3253	1.3082***	1.439	0.8539
QR outcome elasticity Q = 0.75	0.1497	0.6669***	0.3562	1.5593***	2.1543**	1.5829
QR outcome elasticity Q = 0.9	0.2248*	0.7091***	0.2218	1.7806***	1.3774**	1.6361
Change in spend (£M) (numerator)	11.58	58.34	29.11	88.63	43.56	47.50
Change in QALYs (denominator)						
Outcome elasticity [Lomas et al. (5)]	284.34	3,980.74	3,032.94	13,174.35	19,429.79	7,961.48
Outcome elasticity [Hernandez-Villafuerte et al. (15)]	265.41	3,801.00	1,388.22	13,208.54	19,335.15	7,092.99
QR outcome elasticity Q = 0.5	320.71	4,239.87	2,630.58	13,025.87	18,079.74	5,726.91
QR outcome elasticity Q = 0.1	596.40	117.99	2,183.84	8,712.71	10,532.45	5,162.83
QR outcome elasticity Q = 0.25	380.48	2,204.29	1,977.19	11,772.32	16,408.14	3,570.54
QR outcome elasticity Q = 0.75	117.58	7,353.89	2,165.00	14,031.94	24,564.32	6,618.81
QR outcome elasticity Q = 0.9	176.57	7,819.23	1,348.11	16,023.39	15,705.75	6,841.27
Cost-per-QALY (£)						
Outcome elasticity [Lomas et al. (5)]	40,831	15,899	10,524	7,274	2,412	6,432
Outcome elasticity [Hernandez-Villafuerte et al. (15)]	43,612	15,349	20,966	6,710	2,253	6,697
QR outcome elasticity Q = 0.5	36,093	13,760	11,064	6,804	2,409	8,294
QR outcome elasticity Q = 0.1	19,408	494,472	13,328	10,172	4,136	9,201
QR outcome elasticity Q = 0.25	30,422	26,467	14,721	7,529	2,655	13,304
QR outcome elasticity Q = 0.75	98,441	7,934	13,444	6,316	1,773	7,177
QR outcome elasticity Q = 0.9	65,554	7,461	21,590	5,531	2,773	6,943

QALY, quality-adjusted life year; PBCs, Programme Budget Categories.

Data reported in Hernandez-Villafuerte et al. (2022) (15).

Significance levels: *p < 0.05, **p < 0.01; ***p < 0.001.

The clearest pattern of a decreased cost-per-QALY with the mortality rate of the PCT is shown in Figure 2 for cancer. Of note, the PBC cost-per-QALY corresponding to each quantile only changes the estimated outcome elasticity at the quantile. Yet, arguably, the total QALY burden of the PCT also varies and it is larger for PCTs with larger mortality rates. This would magnify the effect of a larger product of outcome elasticities multiplied by larger QALY burdens for PBCs at the upper tail of the mortality rates in cancer and circulatory diseases, making more pronounced the increase in the cost-per-QALY with a decline in the mortality rate. One explanation for the high value of the estimated cost-per-QALY threshold for PCTs in the lower tail of the mortality rate, at quantiles 10% or 25% of the mortality rate in cancer or circulatory diseases may, of course, be that these PCTs are decreasing morbidity more quickly than mortality. We do not know, but it is likely that the surrogacy effects may apply differently for PCTs with low or high mortality. It seems inherently implausible that all PCTs reduce mortality and morbidity at the same rate.

Assuming a 1 SD change in the outcome elasticity for cancer, the York Team (21) estimated 7% variability in the PBC cost-per-QALY or health opportunity costs. Our results from quantile regression show stronger and asymmetric variation of the PBC cost-per-QALY around the central estimates. Martin et al. (16) also estimate outcome elasticities at different quantiles. However, they only present a central threshold with a CI. Our analysis of the results in (15) shows that heterogeneity in the threshold is reflected by an asymmetric interval and with larger variation than that estimated from parametric uncertainty in (5), or around the central estimate in (16). We set out the results in Figure 2 below.

### 4.2. Heterogeneity across clinical areas

Heterogeneity across clinical areas introduces structural uncertainty due to aggregation methods and the assumptions we have discussed in Section 3. Variation by clinical area was used in (4) to give an indication of how sensitive the overall threshold is to the estimate of health effects associated with each PBC. However, the persistence of very large heterogeneity in estimates of the threshold by clinical area as reported in (4, 5) suggest this is a substantial issue relevant to policy making.

We summarise the estimates by clinical area in (4, 5) in Table 6. We have also calculated the implicit PBC cost-per-QALY that results from Martin et al.’s (6) outcome elasticities. These show how the methodological changes in Martin et al.’s (6) regression approach affect the estimation of cost-per-QALY at PBC level. The apparent similarity in the overall thresholds as between the three papers is an artefact of constructing a weighted average where 50% of the NHS budget is allocated to around 70% of QALY change for PBCs with mortality and the other 50% of the budget to 30% of QALY change in PBCs without observable mortality, with weights determined by QALY share.

**Table 6:** Threshold estimates by clinical area in Claxton et al. (4), Lomas et al. (5), and Martin et al. (6).

PBC	Claxton et al. (4) PBC cost-per-QALY (£) 2008/9	Lomas et al. (5) PBC cost-per-QALY (£) 2012/13	Martin et al. (6) PBC cost-per-QALY (£) 2012/13
Cancer	£16,997	£15,899	£5,739
Circulatory	£7,038	£7,274	£10,650
Respiratory	£1,998	£2,412	£4,109
Gastrointestinal	£7,293	£6,432	£12,247
Infectious diseases	£20,829	£40,831	£14,781
Endocrine	£3,124	£10,524	£5,251
Neurological	£5,480	£256,924	£2,312
Genitourinary	£43,813	£707,660	£113,225
Trauma and injuries	N/A	N/A	N/A
Maternity and neonates	£2,969,208	£4,731,851	£250,781
11 PBCs with observed mortality	**£8,308**	**£9,713**	**£5,005**
Disorders of blood	£28,305	£7,189	£8,240
Mental health	£49,835	£14,289	£16,378
Learning disability	£78,854	N/A	£62,466
Problems of vision	£76,850	£41,341	£47,385
Problems of hearing	£19,070	£4,510	£5,169
Dental problems	£55,916	£31,506	£36,112
Skin	£174,775	£84,740	£97,128
Musculoskeletal	£20,254	£13,546	£14,989
Poisoning and adverse events	£163,766	£78,625	£90,123
Healthy individuals	£1,483,012	£346,537	£397,184
Social care needs	N/A	N/A	N/A
Other		N/A	£10,341,523
12 PBCs without observed mortality	** **	**£27,089**	**£34,314**
All 23 PBCs	**£12,936**	**£14,410**	**£9,892**
Elasticity of extrapolation	**0.79**	**1.15**	**1.392**

QALY, quality-adjusted life year; PBCs, Programme Budget Categories.

Claxton et al. (4), York Team (21), and authors’ calculations to estimate implicit results from Martin et al. (6) PBC-level threshold and elasticity of extrapolation.

Bold values mean summary values.

Although the numbers change between the three papers, there is clear evidence of differences in thresholds by disease area. This requires more understanding, as it has important policy implications. This difference by area is reinforced by a similar estimation exercise undertaken in public health, where Martin et al. (27) found that “each additional QALY costs about £3,800 from the local public health budget.”

The detail by clinical area in (4) also reveals, as noted above, that changes in the respiratory PBC had the largest effect on the overall cost-per-QALY threshold. Lomas et al. (5) also find that health opportunity costs are most sensitive to the mortality/morbidity assumption when applied to respiratory PBC. This is because the respiratory PBC accounts for 29.5% of the QALY change and only represents 0.49% of the change in NHS budget (see Supplementary Appendix S1 Table A3). Moreover, 95% of the QALY change in the respiratory PBC is due to QoL during disease, i.e., QALYalive. This makes it very sensitive to the surrogacy assumption. Both (5) and (21) assess sensitivity to the surrogacy assumption, (“health opportunity costs sensitivity to mortality morbidity assumption”) as the percentage of the QALY change from QoL during disease (change in QALYalive) over the total QALY change. As shown in Supplementary Appendix S1 Table A3, in respiratory disease this is 194 QALYs, which is 27.9% of the total QALY change. It is greater than the total of the QALYs generated by all the 12 PBCs without observed mortality data (187 QALYs).

Analysing the sensitivity of the cost-per-QALY threshold to QALY changes coming from the loss of 194 QALYs attributed to QoL during disease in PBC respiratory, Lomas et al*.* (5) estimate an elasticity of the overall threshold to respiratory cost-per-QALY as 3.85, resulting from the 38.5% increase in the overall cost-per-QALY threshold from £14,410 to £19,960. Of note is the non-linearity of this percentage change in the threshold. The growth rate is larger than the related percentage decrease in QALY change, i.e., a 27.9% decrease in QALY change (due to removing pure QoL effects in respiratory diseases) produces a 38.5% increase in overall threshold. In the Supplementary Appendix S1 Table A2, we present calculations that show that use of the QALY burden approach results in a much larger QALY change in respiratory diseases as compared to using the QALY ratio method.

Claxton et al*.* (4) state that the elasticity of the threshold has a linear property, as defined according to the ratio. A proportionate change (increase) in threshold is equal to a proportionate change (decrease) in QALYs, correct up to 50% change in health effects. However, this does not hold. Discrete changes larger than about 20 QALYs (2.9% of total QALY change or total health opportunity costs, i.e., around 3%) produce changes in the cost-per-QALY threshold larger than the percentage change in QALYs.

Considering the sensitivity of the threshold to the mortality/morbidity assumptions, measured as the percentage of change in QALYalive over the total change in QALYs (presented in absolute terms in Supplementary Appendix S1 Table A3), the threshold is sensitive (which we define as a more than a 3% change) to the calculation of the QALY burden with disease (QALYalive) in five PBCs: circulatory, gastrointestinal, endocrine, mental health, and musculoskeletal.

The QALY changes in these PBCs compound the structural uncertainty in estimating outcomes elasticities from different models, and the greater uncertainty introduced by the use of the QALY burden method.

## 5. Discussion and conclusion

We have shown that structural uncertainty related to the assumptions used to move from the outputs of the regression model to an estimate of the cost-per-QALY threshold has a major impact on threshold estimates for the English NHS in the three studies using this approach. In particular,

• the duration assumption of 1 year of effect of health expenditure is essential given the use of a static model to estimate mortality outcome elasticities. A dynamic model would be needed to estimate mortality impacts beyond one year;
• the surrogacy effect requires assumptions relating to the effect of expenditure on morbidity to QALYs for the prevalent and incident population in order to calculate a threshold using the QALY burden. The estimation of the absolute cost-per-QALY threshold is sensitive to these assumptions and methods used;
• the imputation of mortality effects to QoL effects (surrogacy) and to clinical areas without observable mortality (extrapolation) has an important additional effect on the variability of the estimated cost-per-QALY threshold.

We have noted that central estimates presented in (4) may be considered by local decision-makers [e.g., (28)]. However, we have also shown how heterogeneity in both mortality levels (by geography) and in disease areas lead to very different cost-per-QALY thresholds within the overall “average.”

Table 7 summarises the main elements of structural uncertainty and the potential impact on the threshold.

**Table 7:** Summary of structural assumptions to get from mortality estimates to a cost-per-QALY estimate.

Model or evidence gap	Structural assumption	Impact on the threshold quoted in Lomas et al. (5)
Use of a static model	Health effects restricted to one year, and assumed to relate to expenditure in that year	This is consistent with the Lomas et al. (5) threshold. However, both (5) and (6) cite an elicitation exercise reported in (18, 19) as evidence that the threshold is overestimated, as health effects of expenditure last beyond a year. This ignores the counter that today's health depends on past expenditure. Only a dynamic model can capture these effects.
Missing mortality outcomes in 12 PBCs	Extrapolation assumption: assumed equal average proportionate effect as in PBCs with mortality outcomes	Using our extrapolation method, the QALY change decreases by about 70 QALYs, resulting in an increase of threshold from £14,410 to £15,985.
Missing morbidity QALY outcomes for all PBCs	Surrogacy assumption. Two stages. • Assumed perfect surrogacy, i.e., 100% proportionate effect (outcome elasticity) on morbidity QALYs obtained from PBCs with mortality and, from the extrapolation assumption, for the rest of the PBCs • Use of QALY burden rather than QALY ratio approach: absolute QALY burden of disease during 1 year for the population with disease (prevalent and incident) in that year	We illustrate the effect of: • Less than perfect surrogacy. Considering only QALYs from reducing mortality, (i.e. zero surrogacy) increases the threshold from £14,410 to £51,546. This is not appropriate. It is more plausible to expect something in between zero and 100%. For example, if expenditure on QoL improvement has 50% of the impact of that on averting deaths, cost-per-QALY thresholds are between £22,497 and £24,050, depending on the elasticity of extrapolation. • The “QALY burden” method results in a larger QALY change as compared to “QALY ratio method.” The reported impact on the threshold in (4) is to reduce it by 14%.
Missing LYs and QoL evidence for deaths averted	Assumptions that: • Any death averted restores mortality risk to that of the general population (reported as “optimistic”) • The QoL in which these additional LYs are lived is that of the general population rather than the QoL of the original disease (reported as “likely to underestimate” the threshold)	• The structural uncertainty reported in (4) Table 30 restricts health effects to 1 year, which is essential given the static model and not “conservative”. • The “best estimate” of £12,936 uses both the “optimistic” and “underestimate” assumptions. Use of the “conservative” assumption of restricting mortality gain to 2 years leads to a reported threshold of £29,314. • The impact of using disease-specific QoL, rather than general population QoL, is not reported in Table 30. Earlier analysis indicates that it increases the threshold estimate by around 25%.
Small sample size (152 observations) Repeated cross-section sample of 152 observations for each year	Insufficient sample size prevents joint estimation, requiring separate estimation of the model for each PBC, and for each year. In (4, 5), the sum of estimated spend elasticities implies that the sum of change in PBC expenditure for all the 23 PBCs is less than the total assumed change in NHS budget, which means a shortfall of in any change in expenditure. An upward adjustment is applied to all spend elasticities. In (6) model, there is no need to estimate distribution of NHS expenditure across PBCs. The econometric model estimated by GMM can present small sample bias.	The initial papers of Claxton et al. (1, 2) had an estimate of £18,317 revised to £12,936 by the reallocation of the residual change using elasticities $\beta_{3,k}\dot{k}\beta_{3,k}.\;$ In (4) the adjustment implies *k* = *1.38* for 2008–2009. Alternative methods robust to small sample bias have been used for all-causes mortality by (13)
Endogeneity of health expenditure	The choice of instruments can be justified either empirically (socioeconomic instruments) or theoretically as components of the funding rule that defined NHS spend per head. An important implication of the funding rule alternative is the definition of total NHS spend per head as explanatory variable instead of PBC spend per head.	The most important implication is the difference in outcomes elasticities for the same PBCs in each model. This responds to differences between variation of individual PBC spend per head compared to aggregate NHS spend per head. In (5), the QALY change seems to allocate largest share of QALY change for the PBC respiratory, while in (6), this occurs with neurological diseases.
Not reported structural uncertainty arising from heterogeneity	Outcome elasticities are estimated as expected mean effect in a given PBC. This mean effect do not represent patients at the low and high tails of the mortality distribution across health units. Differences in cost-per-QALY thresholds by PBC are reported in (4) but not in (5, 6). Their significance is not discussed.	The use of outcome elasticities estimated using Quantile Regression has important effects. As illustrated for cancer, the threshold could halve at the high mortality tail and more than double at the low mortality tail. We calculate the implied cost-per-QALY thresholds by PBC in (5, 6). Together with (4) numbers they indicate heterogeneity which is arguably relevant for resource allocation and policy making.

QALY, quality-adjusted life year; PBCs, Programme Budget Categories.

We can note that, while we have estimated, in most cases, effects from the main estimates in (4) of £12,936 and (5) of £14,410, the alternative assumptions illustrated in Table 7 can, in principle, be cumulative. For example, if we adjust the “best estimate” in the QALY burden approach from £12,936 to (say) £29,314, this figure would be higher with, for example, different assumptions about the elasticity of extrapolation, and/or about the reallocation of residual expenditures, or if surrogacy was assumed to be less than 100%. There are several sets of plausible structural assumptions that would place the threshold estimates from these studies within the current NICE range of £20,000–£30,000. This does not mean that these assumptions are “right”—they involve judgement calls and, hopefully, stimulate the collection of more evidence. But undertaking and reporting studies of the threshold, without reporting all relevant decision uncertainty, is not serving policymakers well. Any implication that a limited analysis of the parameter uncertainty that can easily be modelled is somehow capturing all uncertainty is also not helpful. Failure to highlight and address structural uncertainty in the analysis and reporting of estimates of health system opportunity cost mean that likely policy relevance, and the willingness of decision-makers to act, is reduced.

The option of seeking to parameterise the structural uncertainty [recommended in (11)] has been undertaken and reported in (18) but has not worked for two reasons. The first, as we have stressed in this paper, is that we have a static model, which impacts the type of questions that can be asked. The second, highlighted in (29), is that there are no experts in the matter at hand. The exercise is, unfortunately, undoable.

A new research agenda is needed to address the structural uncertainties involved in getting from mortality elasticity estimates of incremental NHS expenditures to an estimate of marginal productivity in terms of a cost-per-QALY. The value of research to reduce this structural uncertainty, in terms of its policy impact on decision making, is likely to be high. However, it is not possible to conduct a value of information calculation because we cannot parameterise the structural uncertainty the research would be designed to reduce.

This work could focus on improving estimates of the QALY burden, but, more importantly, would focus on estimating the impact on quality of life of NHS expenditure and ideally derive a separate quality-of-life elasticity by clinical area. As well as increasing the granularity of the data, this would help increase understanding of marginal productivity by disease area. Claxton et al. (4). discuss possible areas of future research, including use of a dynamic rather than a static model; direct measurement of the effects on quality of life by using Patient Reported Outcomes Measures in the disease areas where they are collected, and using quality of life data collected in the treatment of depression and anxiety disorders; and the use of Clinical Practice Research Datalink (CPRD) records to improve understanding of the incidence and duration of disease by age and gender. The priorities for research could be informed by a study using the average per-patient QALY burden from HODaR and MEPS data [as presented in (4)] to identify the sensitivity of the variation in QALY changes for a given change in NHS expenditure to different data and assumptions.

One of the purposes of the Claxton et al*.* (4) study was to establish an approach that could be replicated over time using routinely collected NHS data. However, the 2012 NHS reforms led to centralisation of substantial NHS activity, such that PBC data are no longer available for many areas of expenditure, and it is not possible to link centrally controlled expenditure back to geographical locality and therefore to a corresponding figure of mortality. Thus, tackling structural uncertainty, particularly by research into the estimation of disease burden and into the impact of expenditure on quality of life, will need to be accompanied by a new approach to estimating mortality elasticities by disease area.

As we noted, the evidence of consistent differences in marginal productivity by clinical area is another important area for research and policy consideration. Even if the policy requirement imposed on NICE is to use one “national” threshold, differences by clinical area, if supported by subsequent research, have important implications for either allocative or productive efficiency or both.

The question then arises as to how these estimates in the three studies should be used in the intervening period. Given a model structure of cross-sectional analysis, the mean proportionate effects of health expenditure on mortality as outcome elasticities estimated from the econometric model are robust to different English datasets, different definition of the health location, and different econometric methods applied to same model; for example, similar outcome elasticities from quantile regression at the median in (15) and generalised method of moments (GMM) estimates at the mean in (5). However, model uncertainty—derived from considering NHS spend per person instead of PBC spend per person as health expenditure variable—has important effects on outcome elasticities at the PBC level, and structural uncertainty has not been addressed. Since the reporting of Claxton et al*.* (5), the emphasis has almost exclusively focused on estimating mortality elasticities, rather than quantifying and reducing the uncertainty that arises when translating these into cost-per-QALY thresholds by disease area and for the NHS overall.

Considering these points, we argue that, pending the results of a research agenda, the current econometric model using mortality and health expenditure data should be restricted to assessing the marginal productivity of the NHS in terms of mortality reduction, and, with caution, absolute cost per life year, as reported, for example, in (30). This assessment should be complemented with analysis of efficiency across health locations, in line with Hernandez-Villafuerte et al*.* (15). This could inform an understanding of how the degree of inefficiency affects marginal productivity even without estimating the effect. This could be done using stochastic frontier analysis, where the basic model estimates an overall NHS inefficiency effect that shifts downwards the production function. The alternative of proposing policy changes based on threshold estimates that do not address structural uncertainty, which on plausible alternative assumptions indicate that the “best” estimate of the threshold may be within the current NICE range of £20,000–£30,000, risks a misallocation of NHS resources, reducing overall health gain.

## Data Availability

The original contributions presented in the study are included in the article/Supplementary Material and further inquiries can be directed to the corresponding author.

## Footnotes

^1^On a related note, in supplementary material Appendix 1 Table A1, we analyse the QALY changes estimated in Claxton et al. (4) Table 30 when transitioning from death averted to QALY change. We find an inconsistency of implied average QALY per death averted which results in 8.8 QALYs per death averted instead of 15 QALYs estimated by Claxton et al. for the best “central” estimate. It is not clear what the implications of this are for the threshold, hence we leave the analysis for an Appendix, rather than the main paper.
