# Supplementary material for: The cost-per-QALY threshold in England: Identifying structural uncertainty in the estimates
Source: Front Health Serv. 2023 Jan 19;2:936774. doi: 10.3389/frhs.2022.936774 (PMC10012707; doi:10.3389/frhs.2022.936774)
Supplement: Supplementary file 1 [file Table1.pdf]

SUPPLEMENTARY MATERIAL

THE COST-PER-QALY THRESHOLD IN ENGLAND. Addressing uncertainty in the estimates

B. Zamora and A. Towse

APPENDIX S1. ISSUES ON ESTIMATION OF QALYs

Claxton et al. (2015) (4) progress from using the mortality elasticities to calculate cost per death averted to cost per life year gained to cost-per-QALY for those life years (QALYdeath) and also for expenditure that is purely aimed at improving quality of life (QALYalive), which they calculate using a QALY burden approach.

To understand the difference between YLL and QALY burden, we begin with the cost per death averted threshold (2008) presented in Claxton et al. (2015). Cost per PBC death averted are presented in Appendix 3 Table 163, and shown in column (2) in the Table below. Table 166 shows the cost-per-life year threshold and is shown in column (3) below. The translation from cost per PBC death averted to cost per YLL gives an average implied YLL per PBC death averted equal to 4.53 YLL, shown in column (4). This is consistent with the number reported in Table 166 of ≈ 4.5.

The summary results for QALY threshold estimates using the QALY burden approach are presented in Table 30 and Appendix 3 Table 179, all in (4). In the next Table A1, we show these for the best estimate and for the upper bound. For the best estimate, the numbers are shown in column (5). The implied average QALYs per death averted are shown in column (6). The numbers reported in Tables 30 and 179 in (4) are shown in column (7). For the upper bound estimate, the numbers are shown in column (9). The implied average QALYs per death averted are shown in column (10). The numbers reported in Tables 30 and 179 in (4) are shown in column (11).

It is not clear how this translate to cost per QALY as reported according to an average QALY burden that results in 15 QALYs per death averted, of which 3 QALY are based only on mortality effects (QALYdeath) (see Appendix Table 175 in (4)). Therefore, an excess of 12 QALYs per death averted should result from reducing morbidity (QALYalive). To understand the QALY burden that applies to the calculation of the final cost per QALY threshold, we summarise in Table A1 the operations implied in this translation from cost per death averted to cost per QALY, for the same grouping of PBCs presents in (4): (1) All big four programmes, (2) 11 PBCs with mortality effects, and (3) all the 23 PBCs. We show that the result of average 15 QALYs per death averted is not consistent with the implied YLL per death averted and the estimated cost per YLL. **Instead, we find an average at 8.8 QALYs per death averted.**

SUPPLEMENTARY MATERIAL

THE COST-PER-QALY THRESHOLD IN ENGLAND. Addressing uncertainty in the estimates

B. Zamora and A. Towse

Table A1. Consistency check between cost per YLL and implied average QALYs per death averted

		Best estimate (2008): 1 year effect of expenditure on mortality and ≈4.5 YLL per PBC death averted					Upper bound (2008): 1 year effect of expenditure on mortality and 2 YLL per PBC death averted			
(1)	(2)	(3)	(4)	(5)	(6)	(7)	(8)	(9)	(10)	(11)
PBC grouping	cost per PBC death averted (£) (Claxton et al. Table 163)	cost per life-year (£) (Claxton et al. Table 166)	Implicit number of YLL pre death averted in (Claxton et al. Table 166)	cost per QALY based on QALY burden (£) (Claxton et al. Table 30)	Implied average QALYs per death averted	Tables 30 and 179 Reported average QALYs per death averted	cost per life-year (£) (Claxton et al. Table 166)	cost per QALY based on QALY burden (£) (Claxton et al. Table 30)	Implied average QALYs per death averted	Tables 30 and 179 Reported average QALYs per death averted
(1) All big four programmes	46,692	10,220	4.57 (=46,692/ 10,220	4,872	**9.6**		46,692/**2** ≈23,346	11,040	**4.2**	
(2) 11 PBCs (with mortality)	105,872	23,360	4.53 (= 105,872/ 23,360)	8,308	**12.7**		105,872/ **2** ≈52,936	18,827	**5.6**	
(3) All 23 PBCs	114,272	25,214	**4.53** (= 114,272/ 25,214)	12,936	**8.8**	**≈15.0**	114,272/ **2** ≈57,136	29,314	**3.9**	**≈6.6**

SUPPLEMENTARY MATERIAL

THE COST-PER-QALY THRESHOLD IN ENGLAND. Addressing uncertainty in the estimates

B. Zamora and A. Towse

Table A2. Comparing QALY ratio and QALY burden methods

	**2006 Results: Claxton et al. Tables 119 and 150**				**2008 Results: Claxton et al. Tables 164 and 178**	
		Method QALY ratio	Method QALY burden			Method QALY burden
	Change in PBC deaths	(1) Change in QALYs	(2) Change in QALYs	**(3)=(2)/(1) QALY burden /QALY ratio**	Change in PBC deaths	Change QALY 2008
Cancer	208.03	1699	2121	1.25	213	2064
Circulatory	1237.82	6713	8347	1.24	1,294	8453
Respiratory	1165.14	3215	28,072	8.73	760	17,981
Gastrointestinal	165.42	3605	3922	1.09	149	3441
**All big four programmes**	**2776.41**	**15232**	**42462**	2.79	**2415.44**	**31939**
Infectious diseases	2.59	27	74	2.74	40.97	1229
Endocrine	54.28	2036	6905	3.39	38.29	4749
Neurological	10.59	342	1361	3.98	69	8551
Genitourinary	4.94	12	34	2.83	128	829
Trauma & injuries	0	0	0		0	0
Maternity & neonates	0.24	273	14	0.05	0.32	18
**First 11 PBCs**	**2849.05**	**17922**	**50850**	**2.84**	**2691.27**	**47315**
Disorders of blood	172.87	1087	1215	1.12	215.92	1712
Mental health	3152.02	19,828	10,878	0.55	1874.69	7,469
Learning disability	475.3	2990	207	0.07	108.86	54
Problems of vision	373.19	2348	561	0.24	203.97	333
Problems of hearing	98.72	621	1168	1.88	91.76	1,098
Dental problems	362.72	2282	578	0.25	303.11	533
Skin	162.3	1021	103	0.10	206.34	152
Musculoskeletal	233.59	1469	1005	0.68	380.77	1,819
Poisoning & adverse effects	67.8	426	42	0.10	97.4	64
Healthy individuals	283.09	1781	40	0.02	371.11	53
Social care needs	1043.74	6566	0	0.00	315.43	
Other	0	0	0		0	
**All 23 PBCs**	**9274.39**	**58341**	**66647**	**1.14**	**6860.63**	**60602**

**2006 Results**			**2008 Results**	
1% Assumed change in NHS expenditure				
£678.96 M			£783.98 M	
	Cost per QALY			Cost per QALY
Cost per death averted	based on QALY ratio	based on QALY burden	Cost per death averted	based on QALY burden
£73,208	£11,638	£10,187	£114,272	£12,936

In Table A2, we compare the results based on the QALY burden method with the results using the QALY ratio method. Both methods are based on the same QoL data but they differ in the calculation of YLL.

The fundamental issue in estimating the QALY burden is whether to use or not the YLL associated with the mortality data from the econometric model. If the amount of YLL are considered, QALYs are calculated using the "QALY ratio" method. The so-called "QALY burden" method refers to when the absolute change in QALYs is calculated by directly multiplying the mortality-based outcome elasticities by the QALY burden. Both methods use the same data sources to measure the impact of disease on quality of life norms. This is the Health Outcomes Data Repository (HODaR) supplemented with information from the Medical Expenditure Panel Survey (MEPS). Note that Lomas et al. (2019) only use the QALY burden method in equation. The calculation of $QALY\ gain = \Delta QALY_K$ for each method differs in the use of YLL as specified in the equations below.

QALY ratio method

$$\Delta QALY_K = (Ratio\ QALY\ to\ YLL) * \Delta YLL$$

QALY burden method

$$\Delta QALY_K = \Delta(QALYdeath + QALYalive)$$

Claxton et al. (2015) (4) applies different extrapolation method depending on the method used to calculate QALYs, although this difference is not imposed by data requirements. If QALYs are estimated using an approach the authors call "QALY ratio", then extrapolation considers the ***absolute*** effect of total expenditure on total health gain obtained for the 10 PBCs with observable mortality to the rest of PBCs, that is, it extrapolates the threshold. In contrast, if QALYs are estimated using the QALY burden method, extrapolation considers the effect of total expenditure on the ***relative*** health gain obtained for the 10 PBCs with observable mortality to the rest of PBCs, and extrapolates a weighted average elasticity of extrapolation using outcome elasticities and spend data from the 10 PBCs with observable mortality.

Claxton et al. (4) argue that QALY burden used GBD to calculate YLL which overestimates net YLL as compared with net YLL from ONS used as denominator in QALY ratio. We asses this difference by comparing first QALYs per death averted from both methods (same number of deaths averted used in both methods), and second the ratio between change in QALYs from QALY burden and change in QALYs from QALY ratio for each PBC. These data are presented in (4) for the year 2006 in Table 145 and Table 150.

First, the QALY ratio method results in 6.29 QALYs per death averted, that according to 2006 data on YLL corresponds to 1.52 QALYs per YLG (see Table 145 in (4)). The 2006 results for cost per death averted (£73,208 per death) and cost per QALY based on QALY burden (£10,187 per QALY) imply 7.18 QALYs per death averted and 1.73 QALYs per YLG (7.18 QALYs/4.14 YLL= 1.73). Therefore, a ratio of 1.14 results by dividing the change in QALYs using QALY burden over change in QALYs using QALY ratio: the QALY burden

method results in 14% more QALYs than the QALY ratio method. Also, a 14% difference can be assumed from 2008 data since the information from GBD about incidence (by age and gender) and duration of disease remain unchanged between 2006 and 2008. Considering the implied 8.8 QALYs per death averted that we have shown result from Claxton et al. (4) best estimate of the cost-effectiveness threshold for 2008 a 14% difference results in adding 1.10 QALYs per death averted if using the QALY burden instead of the QALY ratio, which multiplied by the 6860.63 deaths averted result in additional 7,552 QALYs that decrease the cost per QALY threshold in 14%.

When comparing the difference in QALY changes by PBC resulting from the two methods as presented in the column (3) of Table A2, the difference is bigger and puzzling with many more QALYs for the 11 PBCs with mortality data if calculated using the QALY burden method (ratio 2.84) and mainly caused by the large and important difference for Respiratory problems. Only considering the 8.73 ratio of column (3) for this PBC, the excess in QALYs using QALY burden instead of QALY ratio amount to 15,921 QALYs, which has a larger effect on decreasing the threshold by a 36% than the aggregate of 14% for the 23 PBCs. That is, if the QALY burden overestimates the change in QALYs and we consider the QALY ratio instead, the effect on the overall cost per QALY threshold best estimate would result in a best estimate of £17,546 per QALY. The overall effect on the cost per QALY threshold for the 23 PBCs is lower at 14% difference because the QALY ratio method results in more QALYs than the QALY burden method for the PBCs without mortality data, and this is due to the different extrapolation assumptions as applied for either the QALY ratio method —assuming constant PBC cost per QALY as the average of the 11 PBCs with mortality— or for the QALY burden method —assuming a proportional effect based on extrapolated outcome elasticity. Therefore, there is a fundamental methodological and quantitatively important difference which seems to overestimate the QALY change by a factor about 3 for the relevant PBC, these 11 PBCs where the effect of expenditure on mortality can be estimated from the econometric model and surrogated from mortality to QALYs. The difference between the other PBCs without mortality data is due to different extrapolation assumptions, not methodologically sustained, but driven by data availability.

TABLE A3 RESULTS PRESENTED IN LOMAS ET AL. (2019) AND THE RESEARCH PROJECT OF THE YORK TEAM

PBC	Spend elasticity	Outcome elasticity	Change in spend	Change in QALY death	Change in QALY alive	Total change in QALYs	Implied PBC cost per QALY (£) 2012/13
Cancer	1.027	-0.361	£662,898	39	3	42	£15,899
Circulatory	1.285	-1.464	£1,007,028	97	42	138	£7,274
Respiratory	0.928	-1.704	£494,916	11	194	205	£2,412
Gastro-intestinal	0.997	-1.904	£539,721	32	52	84	£6,432
Infectious diseases	0.749	-0.362	£131,519	1	2	3	£40,831
Endocrine	0.951	-0.499	£330,707	2	30	31	£10,524
Neurological	0.856	-0.009	£431,955	0	2	2	£256,924
Genito-urinary	0.855	-0.16	£463,948	0	1	1	£707,660

SUPPLEMENTARY MATERIAL

THE COST-PER-QALY THRESHOLD IN ENGLAND. Addressing uncertainty in the estimates

B. Zamora and A. Towse

PBC	Spend elasticity	Outcome elasticity	Change in spend	Change in QALY death	Change in QALY alive	Total change in QALYs	Implied PBC cost per QALY (£) 2012/13
Trauma & injuries*	1.058	0	£447,083	0	0	0	N/A
Maternity & neonates*	0.833	-0.106	£424,516	0	0	0	£4,731,851
11 PBCs with observed mortality			£4,934,291	182	326	507	£9,713
Disorders of Blood	1.119	N/A	£146,331	1	19	20	£7,189
Mental Health	1.023	N/A	£1,311,251	8	84	92	£14,289
Learning Disability	0	N/A	N/A	0	0	0	N/A
Problems of Vision	1.411	N/A	£369,173	0	9	9	£41,341
Problems of Hearing	1.523	N/A	£78,811	0	17	17	£4,510
Dental problems	0.855	N/A	£348,208	0	11	11	£31,506
Skin	1.158	N/A	£276,256	1	2	3	£84,740
Musculo skeletal	0.725	N/A	£440,253	2	31	33	£13,546
Poisoning and adverse events	1.124	N/A	£125,330	0	1	1	£78,625
Healthy Individuals	1.172	N/A	£242,347	0	1	1	£346,537
Social Care Needs	1.613	N/A	£612,798	0	0	0	N/A
Other	0.585	N/A	£1,114,950	0	0	0	N/A
12 PBCs without observed mortality			£5,065,709	12	175	187	£27,089
All 23 PBCs			£10,000,000	194	500	694	£14,410

APPENDIX S2. ELASTICITY OF THE THRESHOLD

Figure A1. Impact of alternative assumption on the effect of health expenditure on ‘pure Qol’ (change on QALYalive) on the estimate of the threshold

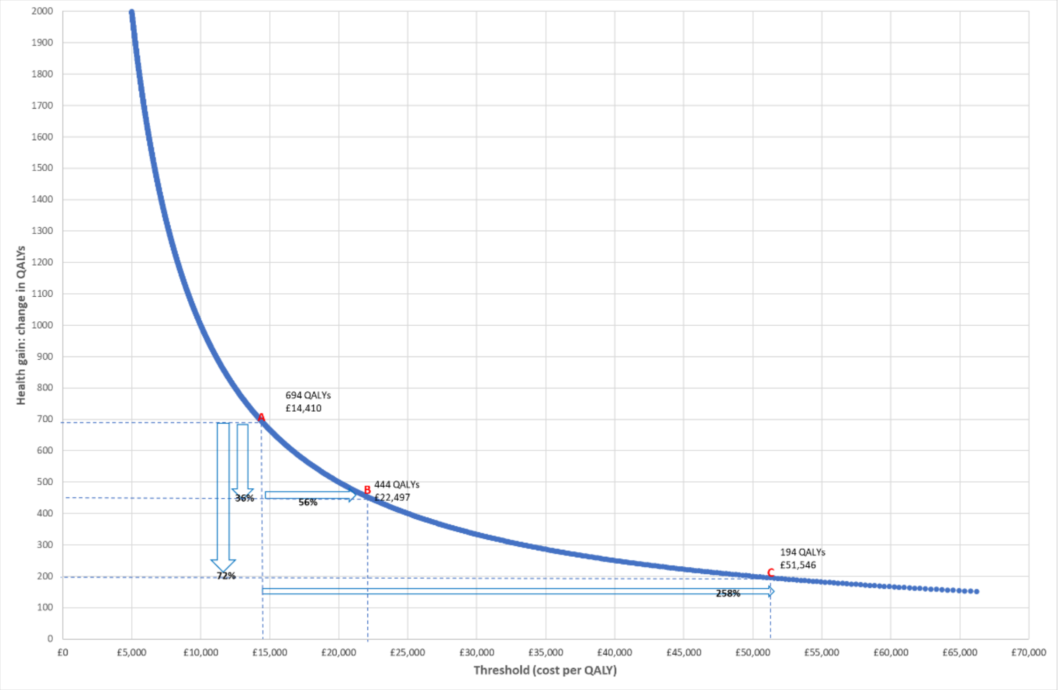

Figure A1 illustrates the change in cost-effectiveness threshold for the effect of £10 million change in NHS expenditure for a range of health effects (change in QALYs).
Point A illustrates Lomas et al. (2019) (5) estimate for 2012/13. Point B results from assuming half health effects on morbidity (QALY alive) than on mortality (QALY death).
Point C assumes there are no effect on improving morbidity during disease (QALY death=0).
The threshold is more sensitive to these assumptions than the change in QALYs.

$$QALY = \frac{1}{K^{\alpha}}\bar{X}$$

Where the parameter $\alpha = 1$, represents the elasticity of the curve:

$$\alpha = \frac{-dQALY/QALY}{dK/K} = 1$$

When the threshold is low, discrete approaches to the elasticity result in elasticities lower than 1. For example, the change from point A to B in Figure A2 implies an elasticity $\alpha = 0.64$.

$$\frac{dQALY}{QALY} = \frac{444 - 694}{444} = 0.36$$

$$\frac{dK}{K} = \frac{22{,}497 - 14{,}410}{14{,}410} = 0.56$$

$$\alpha = \frac{0.36}{0.56} = 0.64$$

However, for larger values of K, α approaches 1.
